# Slipped Capital Femoral Epiphysis Associated With Vitamin C Deficiency in a 7-year-old Boy

**DOI:** 10.5435/JAAOSGlobal-D-21-00012

**Published:** 2021-05-06

**Authors:** Muhammed Nazeer, Rohith Ravindran, Bharat C. Katragadda, Ehsan N. Muhammed, Sanuja Titus, Mohsin N. Muhammed

**Affiliations:** From the Department of Orthopedics, Kerala Institute of Medical Sciences, Anayara, Thiruvananthapuram, Kerala, India (Dr. Nazeer, Dr. Ravindran, and Dr. Katragadda); the Department of Orthopedics, Kasturba Medical College, Mangalore, Karnataka, India (Dr. E. N. Muhammed); the Department of Pediatrics, Kerala Institute of Medical Sciences, Anayara, Thiruvananthapuram, Kerala, India (Dr. Titus); and Kasturba Medical College, Manipal, Karnataka, India (Dr. M. N. Muhammed).

## Abstract

Scurvy is rare in the present world and is mostly found in children with abnormal dietary habits and physical and mental disabilities. Scurvy can present in various forms, mimicking several common diseases, thus making the diagnosis difficult. Spontaneous epiphyseal separation is known to occur in scurvy, although rarely reported. The usual locations of these epiphyseal separations are distal femur and proximal humerus. Our case is unique in that scurvy in a seemingly normal child resulted in proximal femur epiphyseal separation which was not reported previously. We report a case of a 7-year-old boy presenting with pain and swelling in multiple joints for 6 months and later inability to walk. Pseudoparalytic frog-leg posture, dietary history of selective eating, and typical radiologic features made us consider a diagnosis of scurvy which was confirmed by a low serum vitamin C level. He developed epiphyseal separation of proximal femur and was treated with percutaneous screw fixation. Vitamin C supplementation resulted in prompt improvement clinically and radiologically.

Scurvy caused by chronic dietary deficiency of vitamin C is rare in the present world.^1^ This rarity along with heterogeneous nonspecific manifestations makes scurvy a forgotten diagnosis. A high index of suspicion with meticulous history taking, including dietary history, clinical, and radiological features, aids in making the diagnosis.^2-4^

Epiphyseal separation is known to occur in scurvy, although rarely reported. Spontaneous epiphyseal slips of the distal femur, proximal tibia, and proximal humerus have been reported in children with special needs. Vitamin C supplementation and immobilization result in rapid healing and excellent remodeling even in severe slips.^5,6,7,8,9,10,11^

We report a case of proximal femur epiphyseal separation in an otherwise normal child associated with vitamin C deficiency. A review of literature failed to find any previous reference of proximal femur epiphyseal separation secondary to scurvy.

## Case Report

A 7-year-old boy presented to our outpatient department with a history of pain and swelling in multiple joints for 6 months and refusal to walk and sit for 1 month. The child was apparently normal 6 months before, when he had fever for few days documented at 101°F to 102°F, which responded to antipyretics. Subsequently, his parents first noticed swelling of the right ankle followed by bilateral knee swelling associated with minimal pain. The child had lower back pain and bilateral hip pain after few days with painful limping followed by difficulty to sit from a lying position. He was diagnosed to have juvenile spondyloarthropathy and was started on antirheumatic medications. After 6 weeks of using the medications, pain and swellings persisted. He was confined to bed over the past 1 month. The child had recurrent abdominal pain with loss of appetite, which the parents attributed to the effect of medications. He was mainly feeding on milk, juice, and biscuits.

There was no history of trauma, fever, morning stiffness, and small joints involvement. There was no history of skin rash or bleeding manifestations. There was no history of recurrent infections. Birth and vaccination history was insignificant. No behavioral changes were observed. He is a student in the second year of elementary school. There was no family history of similar complaints or any chronic diseases.

On general examination, the child was alert and oriented. Face, hair, and eyes were normal. Mild gingivitis was noted along the upper incisors. No lymphadenopathy and no skin lesions were observed. The body mass index was calculated to be 27.8 kg/m^2^. Respiratory, cardiovascular, abdominal, and neurological examination findings are normal.

The child was lying on the bed with lower limbs in frog-like position and semiflexed at hips and knees. Both knee joints were swollen, warm, and tender. Overlying skin was normal, and thigh muscle wasting was noted. The child was apprehensive and resisted examination of movements at hip and knee joints.

**Figure 1:**
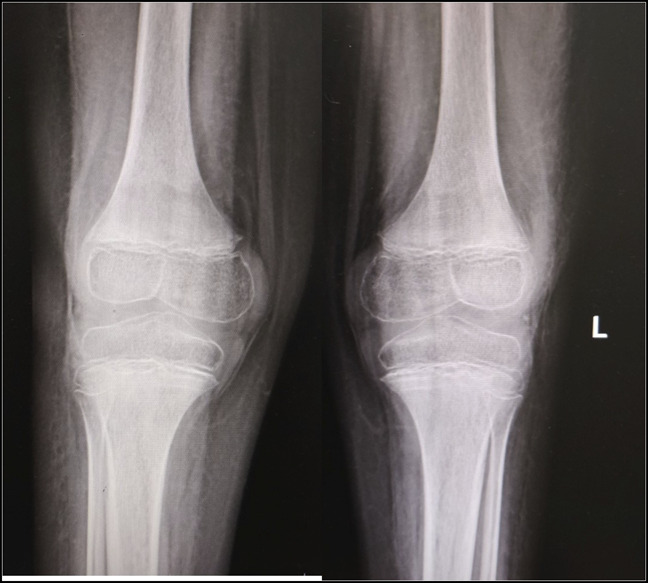
Radiograph of AP of both knees showing osteopenia, ring sign (Wimberger) in epiphysis, radiodense (Frenkel), and lucent (Trummerfeld) lines along the metaphysis and metaphyseal (Pelkan) spurs.

**Figure 2:**
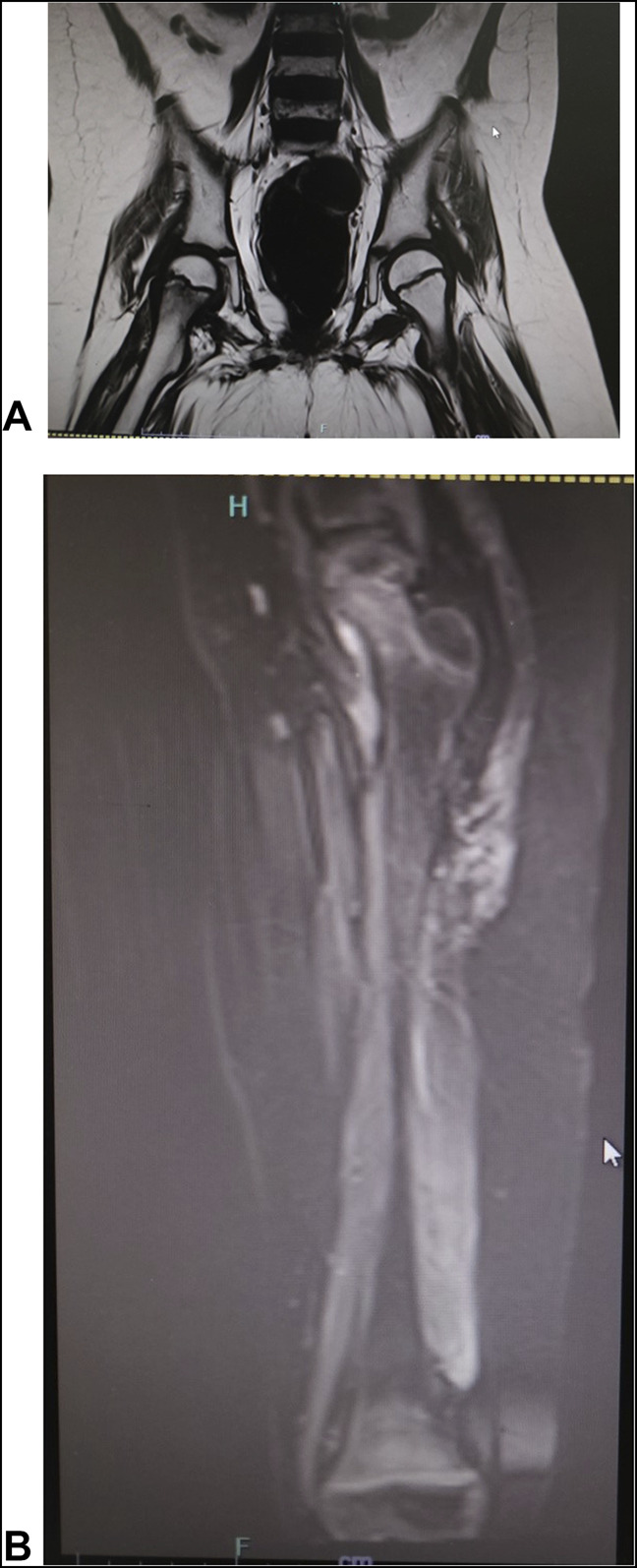
**A**, MRI of both hips showing diffuse marrow edema bilateral proximal femurs, more on the right side. Hip joints are normal. **B**, MRI of left thigh showing diffuse marrow edema along the femur. Thigh and gluteal muscles also show hyperintense signals.

Radiographs of both knees showed signs of osteopenia, thick sclerotic metaphyseal line above a widened physis, and small beak-like excrescences at the metaphysis of both tibiae (Figure 1). Radiograph of pelvis with both hips showed osteopenia. MRI scan of pelvis with both hips showed normal hip joints but diffuse heterogeneous bone marrow signals in the proximal femur (Figure 2, A). MRI scan of the thigh and knee joints showed diffuse marrow edema along the distal femur along with surrounding soft-tissue edema (Figure 2, B).

Hemoglobin was low at 9.1 g/dL (reference range = 11.5 to 15.5 g/dL). Total count, differential counts, reticulocyte count, and platelet count were within normal limits. The erythrocyte sedimentation rate was elevated at 20 mm/hr, but C-reactive protein was normal. Liver function tests and renal function tests were within normal limits. Bleeding time, clotting time, and other tests of bleeding profile were within normal limits. Serum calcium was low at 8.1 mg/dL (reference range = 8.8 to 10.8 mg/dL), whereas phosphate and vitamin D levels were within the normal range. Alkaline phosphatase was elevated to 240 IU/L (reference range = 80 to 180 IU/L). Rheumatoid factor, antinuclear antibodies, antistreptolysin O titer, and HLA-B 27 were negative. Serum iron was low at 24 μg/dL (range = 59 to 158 μg/dL), whereas copper and zinc levels were normal.

Ultrasonography abdomen showed normal study. Endoscopy showed ulcers in the fundus and body of the stomach. Nasojejunal feeds were started because the child was not tolerating oral intake.

Based on pseudoparalytic posture, dietary history suggestive of selective food intake, and with typical radiological features, a diagnosis of scurvy was considered. Serum vitamin C evaluation was done, which showed low levels of 0.8 mg/L (reference range = 2 to 14 mg/L).

**Figure 3:**
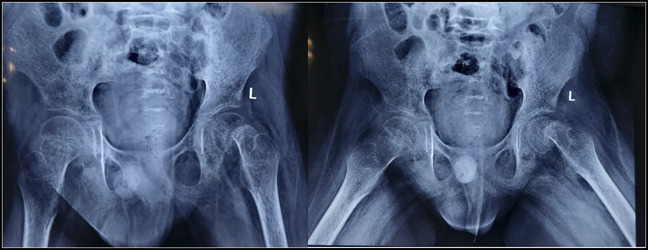
Radiograph of the pelvis with both hips of AP view and frog-leg lateral view showing left the.

**Figure 4:**
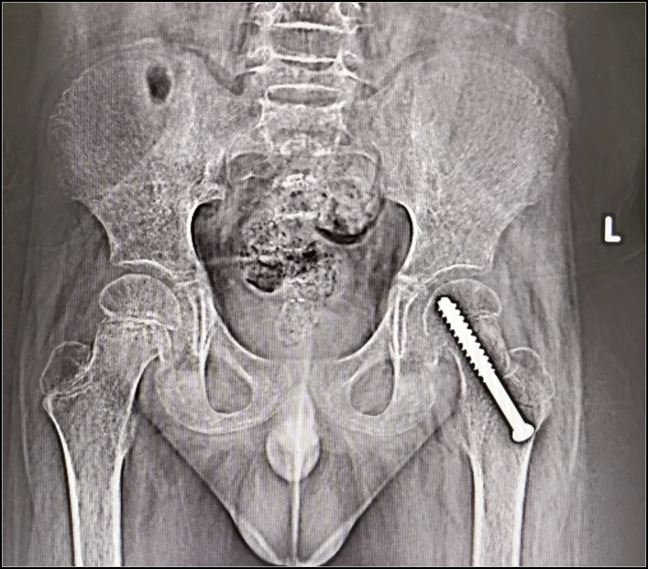
Radiograph at the 6-month follow-up showing screw in situ and no evidence of slip progression and osteonecrosis.

Thus, a diagnosis of scurvy was made, and the child was started on vitamin C supplementation 500 mg/d in two divided doses. Bilateral skin traction was applied to avoid contractures after which pain was reduced, and he was mobilized as tolerated after 1 week. He had sudden severe pain in the left hip. Radiographs showed epiphyseal separation of the proximal femur (Figure 3). Endocrine workup was done in view of slipped epiphysis, which showed normal thyroid, parathyroid, cortisol, insulin, and growth hormone levels. In the surgical room, gentle manipulation reduced the epiphyseal slip and percutaneous screw fixation was done. Vitamin C therapy was continued at 500 mg/d for 2 weeks followed by 250 mg/d for the next 2 months. Folic acid, vitamin B complex, calcium, and vitamin D supplementation were continued. At the 2-month follow-up, his pain had resolved, and he was ambulating normally. His appetite improved and started taking normal diet. After 6 months, the child was walking well with no residual deformity clinically and radiologically. Radiographs of both hips showed no evidence of slip progression, osteonecrosis, or screw-related complications (Figure 4).

## Discussion

A young child with bone and joint pains with refusal to walk usually alerts the orthopaedic surgeon to a diagnosis of juvenile idiopathic arthritis, osteomyelitis, and septic arthritis. Bone pains with bleeding manifestations may point to acute leukemia. Rickets should be considered in a child from a low socioeconomic group. Scurvy is often forgotten during evaluation because of the rarity and the polymorphisms of the clinical signs and symptoms.^2,3^

Scurvy is rarely seen in present world because of improvement in nutritional and socioeconomic status of most populations. Children with feeding problems and physical and mental disabilities are prone to deficiency,^4^ 8 to 12 weeks of inadequate intake of vitamin C can result in clinical symptoms. Initial symptoms are vague and include decreased appetite, irritability, and myalgias. Later, classic scorbutic manifestations such as gingivitis, petechiae, arthralgias, hemarthrosis, and subperiosteal hemorrhages occur.

Radiological features manifest after 3 to 6 months of nutritional deficiency. Typical features include sclerotic rim around epiphysis (Wimberger ring sign), dense provisional calcification zone (white line of Frenkel), adjacent lucent band (Trummerfeld zone), and small metaphyseal corner fractures (Pelkan spurs).^1^ MRI scan findings are nonspecific and include bilateral symmetric metaphyseal heterogeneous signal changes, subperiosteal fluid, and adjacent soft-tissue edema.^12^

Partial or complete separation of epiphysis is known to occur in scurvy, although rarely reported.^5,6,7,8,9,10,11^ The basic biochemical defect in scurvy is failure to hydroxylate proline and lysine, an essential step in collagen formation. In the physis, the palisade of cartilage cells is formed as usual, but the osteoblasts fail to lay down osteoid matrix and resorption of calcified cartilage is slowed. This results in a loose, disorganized connective tissue, and the stress of weight bearing or muscle tension may result in physiolysis. Separation of the epiphysis always occurs through the zone of calcified cartilage, which in the radiographs is represented as the white line of Frenkel. The poorly formed capillaries rupture easily, and the loosely attached periosteum leads to an extensive subperiosteal hematoma, which also predisposes to epiphyseal separation.^7^

The usual locations of these epiphyseal separations are distal femur, proximal tibia, and proximal humerus. Separation of proximal femur epiphysis was not reported previously. Previous literature recommends managing the epiphyseal separations conservatively by splinting and observation. On nutritional supplementation, the lesions heal and remodel well because there is an intact periosteal sleeve, and the epiphyseal separation occurs through a relatively avascular zone of provisional calcification, causing little or no damage to the epiphyseal or metaphyseal blood vessels. Closed or open reduction of the slip is rarely required, and complete recovery with remodeling is the rule.^6,10^ We opted for gentle manipulation and percutaneous screw fixation to prevent future femoroacetabular impingement and hip osteoarthritis.^13^

## Conclusion

Vitamin C deficiency can lead to epiphyseal separation in some children with abnormal dietary habits. Proximal femur epiphyseal separation can also occur, which was not reported previously. Vitamin C supplementation and percutaneous screw fixation resulted in good healing without complications.

A high index of suspicion with stress on detailed history taking, including dietary history, clinical, and radiological findings, aids in making a diagnosis of scurvy. Low serum vitamin C levels and prompt response to vitamin C therapy confirm the diagnosis. Early diagnosis avoids notable morbidity and avoids costly, more invasive diagnostic tests.

## References

[1] Agarwal A Shaharyar A Kumar A Bhat MS Mishra M: Scurvy in pediatric age group—A disease often forgotten? J Clin Orthop Trauma 2015;6:101-107. 25983516 10.1016/j.jcot.2014.12.003 PMC4411344

[2] Naranje S Kelly DM Sawyer JR: A systematic approach to the evaluation of a limping child. Am Fam Physician 2015;92:908-916. 26554284

[3] Alqanatish JT Alqahtani F Alsewairi WM Al-kenaizan S: Childhood scurvy: An unusual cause of refusal to walk in a child. Pediatr Rheumatol Online J 2015;13:23. 26063195 10.1186/s12969-015-0020-1 PMC4462115

[4] Ratanachu-Ek S Sukswai P Jeerathanyasakun Y Wongtapradit L: Scurvy in pediatric patients: A review of 28 cases. J Med Assoc Thai 2003;86(suppl 3):S734-S740. 14700174

[5] Scott W: Epiphyseal dislocations in scurvy. J Bone Joint Surg Am 1941;23:314-322.

[6] Silverman FN: Recovery from epiphyseal invagination: Sequel to an unusual complication of scurvy. J Bone Joint Surg Am 1970;52:384-390. 5440019

[7] Nerubay J Pilderwasser D: Spontaneous bilateral distal femoral physiolysis due to scurvy. Acta Orthop Scand 1984;55:18-20. 6702422 10.3109/17453678408992304

[8] Quiles M Sanz TA: Epiphyseal separation in scurvy. J Pediatr Orthop 1988;8:223-225. 3350960

[9] Hosalkar HS Johnston DR Pill S Flynn JM: Multiple epiphyseal separations in a child with scurvy and cerebral palsy. Am J Orthop (Belle Mead NJ) 2005;34:295-298. 16060558

[10] Aroojis AJ Gajjar SM Johari AN: Epiphyseal separations in spastic cerebral palsy. J Pediatr Orthop B 2007;16:170-174. 17414775 10.1097/01.bpb.0000192057.68058.76

[11] Gupta S Kanojia R Jaiman A Sabat D: Scurvy: An unusual presentation of cerebral palsy. World J Orthop 2012;3:58-61. 22655223 10.5312/wjo.v3.i5.58 PMC3364318

[12] Gulko E Collins LK Murphy RC Thornhill BA Taragin BH: MRI findings in pediatric patients with scurvy. Skeletal Radiol 2015;44:291-297. 25109378 10.1007/s00256-014-1962-y

[13] Hosalkar HS Pandya NK Bomar JD Wenger DR: Hip impingement in slipped capital femoral epiphysis: A changing perspective. J Child Orthop 2012;6:161-172. 23814615 10.1007/s11832-012-0397-z PMC3399996

